# What works for whom in pediatric OCD: description of causally interpretable meta-analysis methods and report on trial data harmonization

**DOI:** 10.1017/S0033291724003301

**Published:** 2025-02-06

**Authors:** Lesley A. Norris, David H. Barker, Ariella R. Rosen, Joshua Kemp, Jennifer Freeman, Kristen G. Benito, Project Harmony Team

**Affiliations:** 1 Department of Psychiatry and Human Behavior, Brown University Warren Alpert Medical School, Providence, RI, United States; 2 Pediatric Anxiety Research Center at Bradley Hospital, East Providence, RI, United States

**Keywords:** causally interpretable meta-analysis, dissemination, harmonization, individual participant data, pediatric OCD, translation

## Abstract

**Background:**

Improving patient outcomes will be enhanced by understanding “what works, for whom?” enabling better matching of patients to available treatments. However, answering this “what works, for whom?” question requires sample sizes that exceed those of most individual trials. Conventional methods for combining data across trials, including aggregate-data meta-analysis, suffer from key limitations including difficulty accounting for differences across trials (e.g., comparing “apples to oranges”). Causally interpretable meta-analysis (CI-MA) addresses these limitations by pairing individual-participant-data (IPD) across trials using advancements in transportability methods to extend causal inferences to clinical “target” populations of interest. Combining IPD across trials also requires careful acquisition and harmonization of data, a challenging process for which practical guidance is not well-described in the literature.

**Methods:**

We describe methods and work to date for a large harmonization project in pediatric obsessive-compulsive disorder (OCD) that employs CI-MA.

**Results:**

We review the data acquisition, harmonization, meta-data coding, and IPD analysis processes for Project Harmony, a study that (1) harmonizes 28 randomized controlled trials, along with target data from a clinical sample of treatment-seeking youth ages 4–20 with OCD, and (2) applies CI-MA to examine “what works, for whom?” We also detail dissemination strategies and partner involvement planned throughout the project to enhance the future clinical utility of CI-MA findings. Data harmonization took approximately 125 hours per trial (3,000 hours total), which was considerably higher than preliminary projections.

**Conclusions:**

Applying CI-MA to harmonize data has the potential to answer “what works for whom?” in pediatric OCD.

Pediatric obsessive-compulsive disorder (OCD) is a debilitating condition that interferes with youth educational, social, and emotional development (e.g., Piacentini et al., 2003; Valderhaug & Ivarsson, 2005). Recent meta-analyses provide strong support for clinically meaningful reduction in symptoms using cognitive behavioral therapy (CBT) with exposure and response prevention (ERP) and pharmacological treatment options (Cervin et al., 2024; Chorpita & Daleiden, 2009; Freeman et al., 2018; Öst et al., 2016). However, a significant number of patients do not show clinically meaningful symptom reduction (McGuire et al., 2015). In addition, although previous meta-analyses have provided important insights about potential predictors and moderators (see Figure 1 for an overview), findings remain inconsistent across studies. To further optimize outcomes for youth with OCD, more work is needed to tailor the selection of existing treatments or treatment combinations to the individual.

**Figure 1.**
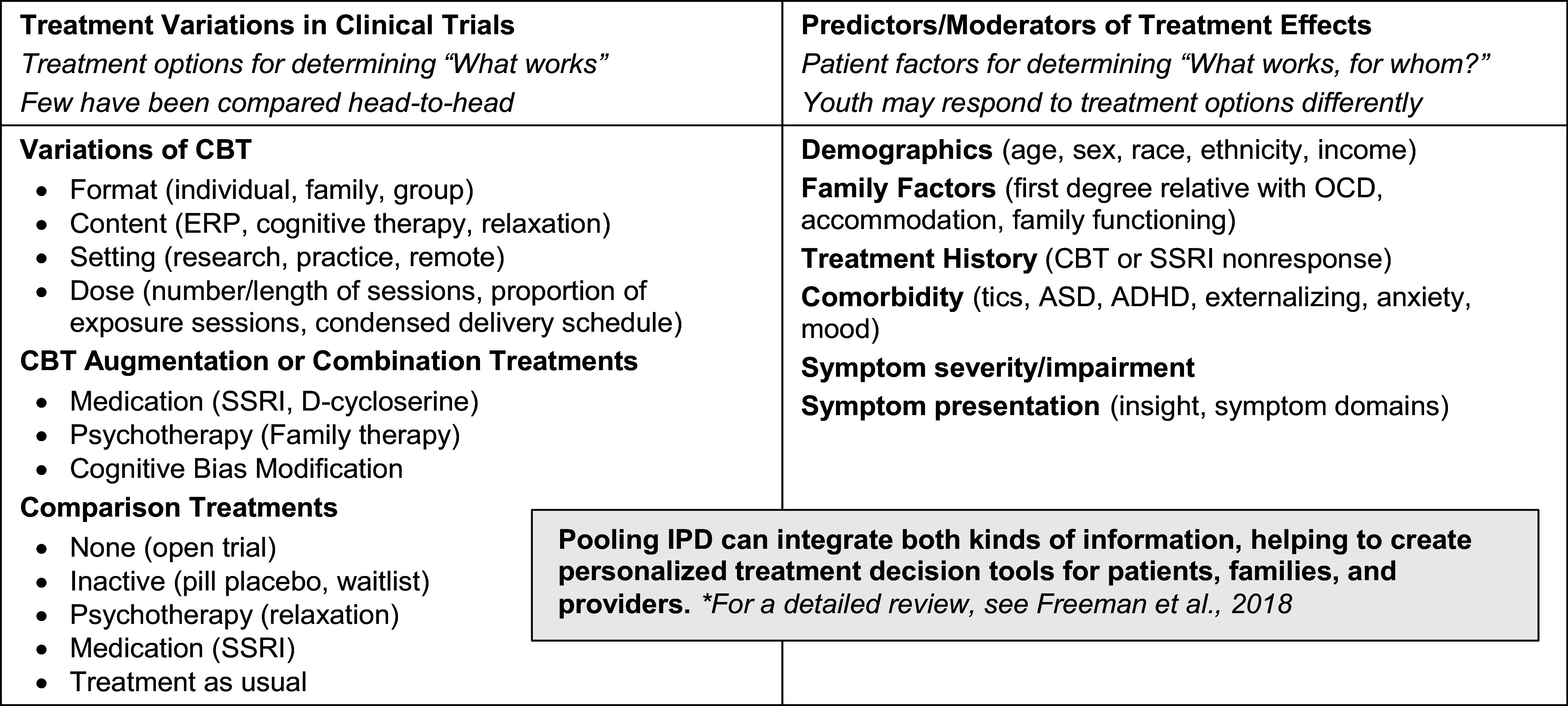
Preliminary factors for inclusion in IPD-MA analysis based on previous meta-analytic work.

Answering this “what works for whom?” question requires comparative effectiveness estimates (what works) in clinically meaningful subpopulations (for whom). The standard approach to generating such estimates would involve running numerous randomized controlled trials (RCTs) comparing each available intervention in each subpopulation. Such time- and resource-intensive studies are prohibitive. The next best option is to use a patchwork of existing trial evidence to conduct meta-analyses. Meta-analytic work to date has identified several patient characteristics that are associated with better outcomes, including younger age, lower symptom severity, lower functional impairment, and better family functioning (Kemp et al., 2021; McGuire et al., 2015; Turner et al., 2018). However, conflicting findings across different meta-analyses highlight significant barriers to evidence synthesis using aggregate data meta-analytic approaches. For example, one meta-analysis suggests that better outcomes are no longer associated with younger age when trials of the youngest children (ages 3–8) are removed (Öst et al., 2016). Age and treatment response are thus confounded by variation in treatment approach (e.g., high family involvement in treatment for young children) and sample characteristics across trials; these relationships cannot be disentangled via conventional aggregate-data meta-analysis. Findings related to comorbidity are similarly mixed and may depend on comparison conditions used per trial. For example, McGuire et al. (2015) report improved CBT outcomes for youth with comorbid anxiety (only in trials with nonactive comparison) and youth with tic disorders (only in trials with active comparison). However, a more recent systematic review suggests attenuated CBT outcomes with the presence of any comorbidity (Kemp et al., 2021).

Inconsistent findings highlight important limitations of conventional aggregate-data meta-analysis in answering “what works, for whom?” One key limitation relates to implementation differences across trials, comparisons of which have been described as “apples to oranges” (Freeman et al., 2018). Individual studies have tested a variety of different CBT formats, content, and doses, in a variety of settings, and using different comparison conditions. Studies have also tested a variety of CBT augmentation treatment strategies, such as serotonin reuptake inhibitors (SRIs; e.g., clomipramine, sertraline, fluoxetine), other medications (e.g., d-cycloserine), and family therapy. Few of the above options have been directly compared in the same trial. To make such comparisons using existing trial data across studies requires making causal assumptions. Conventional meta-analysis does not have a clear causal interpretation when the distribution of effect modifiers differs among the populations underlying the included trials and the target population. Results of each trial apply to the population underlying it (reflecting the trial’s eligibility criteria and recruitment practices), and that population typically has a different distribution of effect modifiers than treatment populations (Dahabreh et al., 2020; Pearl & Bareinboim, 2014). Conventional meta-analytic methods do not address these concerns.

Causally interpretable meta-analysis (CI-MA) pairs individual-participant-data (IPD) from trials and data from target populations using recent advancements in transportability methods to address limitations of conventional aggregate-data and IPD meta-analysis (see Table 1 for an overview). Transportability methods account for differences between the population underlying a trial and the target population by combining background knowledge, causal assumptions, statistical methods, data from the trials, and a sample of baseline characteristics from a target population to extend causal inferences from the trials to that target population. Robust statistical models are used to address between-trial differences in covariates, enabling the transportation of causal estimates from trial samples to target populations. Under explicit causal and statistical assumptions, which are weaker than the implicit assumptions required by conventional meta-analysis, the transported analyses provide unbiased estimates of how interventions will fair in the target population(s), enabling “apples-to-apples” comparisons of interventions even when two interventions have not been compared directly in a head-to-head trial. In addition, the target population can be defined as subpopulations, enabling evaluation of relevant interventions for those subpopulations. Importantly, this can include information about minoritized subpopulations, who have been historically underrepresented in OCD trial samples (e.g., 91% White and 99% non-Latinx in a systematic review of adult trials; Williams et al., 2010). Thus, CI-MA represents a powerful advance in evidence synthesis and may better facilitate understanding “what works, for whom?” This approach is critically important for pediatric OCD treatment personalization but also has far-reaching implications for other conditions where low within-trial sample sizes hamper efforts to identify treatment predictors/moderators with greater specificity.

**Table 1.** Benefits of transportability methods

	MA	IPD-MA	IPD-MA-T
**Required assumptions**			
Within trial identification conditions (3 assumptions)	–	–	–
Target population identification conditions (3 assumptions)			–
**Capabilities**			
Analysis of subpopulations	–	–	–
Assumptions conditional on covariates		–	–
Accommodates non-randomized designs		–	–
Novel treatment comparisons		–	–
Uses information from target populations			–
Inferences in populations that differ from trials			–
Novel treatment comparisons in subpopulations			–

*Note.* MA, meta-analysis; IPD-MA, individual participant data meta-analysis; IPD-MA-T, transportability analysis.

Despite the promise of CI-MA methods, they are under-utilized. This is in part because CI-MA requires well-curated IPD, including detailed records of trial design and implementation, as well as a detailed accounting of the intervention (Barker et al., 2022). While most investigators meet the current requirements of funding agencies to make data available upon request, there continues to be great variability in the quality of documentation provided because it is costly and time-intensive to integrate datasets and documentation into archival-quality packages that meet modern standards of reproducible research. In addition, IPD needs to be harmonized to a common template (i.e., same variable names, value labels, scoring procedures). Principal investigators are best positioned to harmonize their data, but it is only recently that investigators have begun allocating appropriate funds to generate well-curated IPD data. Moreover, the majority of CBT trials in pediatric OCD occurred prior to federal data-sharing requirements; data for these have never been harmonized and are not publicly available. These seminal trials established the efficacy of CBT for pediatric OCD, and trials of this nature will not be repeated in the future. As such, harmonizing these trial data would provide a strong, unparalleled repository of evidence from which to examine treatment response in this and future studies.

Any answers that emerge from CI-MA studies are also at risk of falling through the “leaky pipeline” from research to translation into clinical practice. Unlike RCTs, which provide group-level estimates of what works for patients on average, results from CI-MA studies have the potential to more directly inform clinical decision-making for individual patients. Knowledge about which treatments work for whom could also allow for more efficient leveraging of limited available resources (i.e., offering the most effective treatment to the individual from the beginning of their treatment course). However, this potential clinical utility cannot be tapped without a strong grounding in dissemination and implementation considerations from the project’s outset. An integrated knowledge translation model (Grimshaw et al., 2012; Kothari, McCutcheon, & Graham, 2017) suggests that effective dissemination and implementation efforts must involve partnerships with key end-users (i.e., clinicians, parents, patients, and advocates) who can act on study findings. Ideally, partners are involved in each project step and share the power to select meaningful outcomes, interpret analyses, and translate findings into accessible formats for other end users. Indeed, emerging evidence suggests the authentic engagement of partners as full participants in all phases of a research project can enhance the quality and resulting impact of findings (Woolf et al., 2016). However, to date, few IPD studies have involved key partners at any stage of the research process.

Here we describe harmonization and CI-MA methods for Project Harmony, which will acquire and harmonize individual patient data from 28 RCTs of youth OCD treatments (*N_expected_* = 1,900), along with target data from treatment-seeking youth and young adults ages 4–20 with OCD entering the intensive and outpatient treatment programs at Bradley Hospital’s Pediatric Anxiety Research Center (PARC) and the outpatient program at the University of South Florida Rothman Center (USF; *N_expected_* = 853), to generate the largest harmonized IPD dataset available to date for pediatric OCD trials. Of note, expectations around the time required for harmonization projects vary widely across reviewers, funders, researchers, and other decision-makers. As misaligned expectations can be a significant barrier to obtaining funding and completing harmonization projects, we report an estimate of approximate person-hours required to date for data acquisition and harmonization. Following data harmonization, a process that is still underway for the current project, comparative effectiveness estimates will then be generated in the target population. Estimates will also be used to compare treatment effectiveness in subsamples of the target population. Subgroup constructs of primary interest were selected *a priori* to overlap with those examined in previous meta-analyses of OCD treatment trials (i.e., demographics, family factors, treatment history, comorbidity, OCD symptom severity/impairment, and global functioning/disability). Data-driven approaches to specific variable selection will also be used. In addition, treatment variations in clinical trials will be examined, including CBT variations (i.e., format, content, setting, dose), CBT augmentation or combination treatments, and comparison treatments (i.e., open trial, inactive, psychotherapy, medication, and treatment as usual). Variables for treatment variations will be selected using both a theoretical and data-driven approach involving detailed coding of each trial’s treatment manual by two independent coders. All de-identified harmonized trial data and syntax will be made publicly available through the NIMH Data Archive (NDA). Throughout the study, the research team will work to disseminate findings through an ongoing strategic partnership with the International OCD Foundation (IOCDF).

## Methods

### Overview

**Figure 2.**
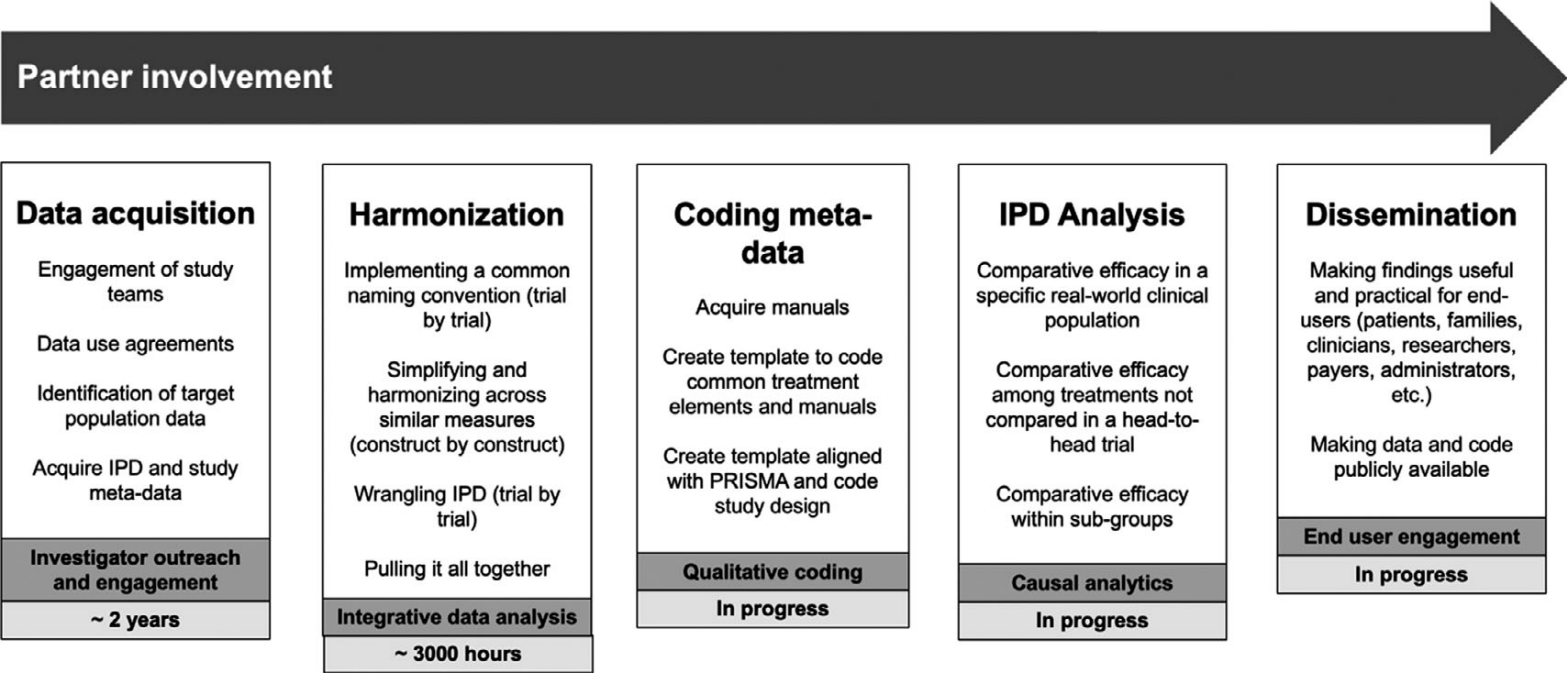
Phases of Project Harmony.

We describe five study phases: (1) data acquisition, (2) harmonization, (3) coding meta-data, (4) IPD analysis, and (5) dissemination (Figure 2) and detail partner involvement that occurred throughout the project.

### Partner involvement

A group of Dissemination Partners (*N* = 8) was assembled at the project outset to represent the interests of end users and ensure that study findings are made accessible and actionable. Partners consisted of patients, parents, pediatric OCD clinicians, and leadership/advocacy representatives from IOCDF. The group will meet bi-annually during: (1) Pre-harmonization, (2) Post-harmonization/Pre-analyses, and (3) Post-analyses/Dissemination. Discussions will focus predominantly on the dissemination of findings (see “Dissemination” section).

### Data acquisition

The Project Harmony team completed a one-year PCORI planning grant to build consensus for an IPD-MA in pediatric OCD. This resulted in commitments from PIs representing 28 RCTs for pediatric OCD treatments (*N_expected_* = 1,900) identified using a recent evidence-based update (Freeman et al., 2018) and an updated search for this project using the same search strategy. Specifically, we searched Medline and PubMed (keywords: obsessive-compulsive disorder OR obsessive behavior; exposure therapy OR behavior therapy OR cognitive-behavior therapy OR treatment; AND children OR adolescents OR pediatric). Studies were included if they (1) involved a sample size >30 (including open trials), (2) focused on children and adolescents 18 years old and younger, (3) required participants to have a primary or co-primary diagnosis of OCD, and (4) reported OCD outcome measures. Studies were excluded if they were (1) non-treatment studies, (2) psychopharmacology interventions only, (3) secondary analyses, (4) case studies, or (5) reviews. Following the primary search, we conducted a hand search of the reference sections from recent meta-analyses, review articles, and studies identified through the primary search. Finally, in some instances, PIs who committed larger trials also volunteered data from smaller trials that they were able to access; thus, there are a few included studies with sample sizes less than the threshold (N > 30) we set with the systematic search. Trials represent the core of currently available evidence for behavioral pediatric OCD interventions (see Table 2 for a summary).

**Table 2.** Trials included in the harmonized dataset

Authors	Trial Type	CBT format (Augmentation)	*N*
Aspvall et al. (2021)	RCT	Individual	152
Farrell et al. (2010)	Open	Family-based individual/group (effectiveness)	35
Farrell et al. (2012)	Open	Family-based group (effectiveness)	43
Farrell et al. (2013)	RCT	Family-based individual (DCS)	17
Farrell et al. (2016)	Open*	Individual (effectiveness)	10
Farrell et al. (2022)	RCT	Individual (DCS)	100
Farrell et al. (2022)	RCT	Family-based individual (DCS)	46
Franklin et al. (2011)	RCT	Individual	124
Freeman et al. (2008)	RCT	Family-based individual	42
Freeman et al. (2014)	RCT	Family-based individual	127
Lenhard et al. (2017)	RCT	Non-face-to-face	67
Lewin et al. (2014)	RCT	Family-based individual	31
Mataix-Cols et al. (2014)	RCT	Individual (DCS)	27
O’Neill et al. (2017)	RCT	Individual	49
Peris et al. (2017)	RCT	Family-based individual (PFIT)	62
Piacentini et al. (2002)	Open	Individual	42
Piacentini et al. (2011)	RCT	Family-based individual	71
Piacentini et al. (in recruitment)	RCT	Individual (App-enhanced)	32
POTS Team (2004)	RCT	Individual	112
Skarphedinsson et al. (2015)	RCT	Family-based individual (effectiveness)	50
Storch et al. (2007)	RCT	Family-based individual	40
Storch et al. (2010b)	RCT	Individual	30
Storch et al. (2010a)	Open	Family-based individual	30
Storch et al. (2011)	RCT	Non-face-to-face	31
Storch et al. (2013)	RCT	Individual	47
Storch et al. (2016)	RCT	Family-based individual (DCS)	142
Torp et al. (2015)	Open	Family-based individual (effectiveness)	269
Turner et al. (2014)	RCT	Non-face-to-Face	72
		Total	1,900

*Note*: CBT, Cognitive Behavioral Therapy; DCS, D-Cycloserine; Effectiveness, Testing efficacy in community setting; Non-Face-to-Face, Internet or phone delivered CBT; Open, Open Trial; PFIT, Positive Family Interaction Therapy; RCT, Randomized Controlled Trial.

Following preliminary outreach efforts, data-sharing agreements were acquired following the policies of each original trial’s home institution; timelines to procure data-sharing agreements varied, taking up to several months per institution (~18 months total). Once acquired, PIs of original trials provided data dictionaries, de-identified item-level IPD including all measures available (see Table 2 for overlapping measures), and treatment manuals for each trial.

To date, we have received 26 out of an expected 28 data dictionaries and 19 datasets. We are in the process of obtaining five additional datasets. The remaining four studies are international and require alternative methods for analyzing their data on-site due to restrictive data-sharing regulations.

### Harmonization

The goal of harmonization is to put variables used across trials on the same metric. This is a multi-step process that includes renaming variables, using logic to equate response options, using normative data to equate measures with clinical interpretations (e.g., dichotomizing using clinical cut-offs, centering variables using clinical cut-offs, standardizing using minimal clinically important difference), and implementing complex latent variable measurement models accounting for between-trial measurement differences. Latent variable approaches to harmonization (e.g., integrative data analysis; Curran & Hussong, 2009) are typically performed on outcomes and not predictors or covariates. For the purposes of this project, the Children’s Yale-Brown Obsessive-Compulsive Scale (CY-BOCS; Scahill et al., 1997) will be the primary outcome, as it has the advantage of being assessed in all trials (Table 2), is continuously distributed, and has clear, clinically meaningful, benchmarks. We will also explore other outcomes, including global improvement, as assessed using the Clinical Global Impressions - Improvement (CGI-I; Guy, 1976) scale. As both measures were assessed in all trials, we will not be implementing latent variable approaches to harmonization in this study and will thus focus this manuscript on the pragmatic process of ensuring common variable names, variable labels, response options, and value labels. While this process is not particularly innovative, the pragmatic process is needed in studies using harmonized data and there is minimal documentation about how much effort is required to complete these steps. The harmonization of target population data is not reflected in the time estimates below. Harmonization was directed by a clinically trained data scientist (DHB) and implemented by a bachelor’s-level research assistant (ARR), with clinical perspective and consensus input provided by a leader in OCD research (KGB). A clinical postdoctoral fellow (LAN) provided additional consensus.

#### Implementing a common naming convention (trial by trial)

Collectively, data dictionaries (*n* = 26) contained 55,396 unique variable names. The number of unique variable names per data dictionary ranged from 79–21,984; the higher number is attributable to having item-level data with unique variable names for each assessment point. Data dictionaries were restructured so that each item had the same unique name across assessments. A common naming convention was then applied to restructured variables (*n* = 18,896) and a crosswalk was created from each trial to the naming convention. This process took around 22 hours (range 8–40) for studies that included item-level variables (*n* = 22) and 3 hours (range 2–3) for studies that did not (*n* = 4). Time varied according to the number of variables, consistency in naming convention, data dictionary format, document type (e.g., SPSS, Word, PDF), completeness of the dictionary (e.g., missing variable labels or response options, unidentified measure versions), and number and complexity of survey-style questions relating to demographics and family/medical history. The total time for this step was ~436 hours, predominantly done by ARR, guided and trained by DHB.

#### Implementing common value labels and responses (measure by measure)

At the end of this first pass, the total number of unique variables was 18,896; 1,437 focused on treatment processes (i.e., assessed during the course of treatment) and were set aside to be harmonized later, leaving 17,459 for further harmonization. We used a relational database to map trial-level variables (variable names, labels, response options, etc.) onto a shared table of harmonized names, using variable names as a key to link various tables within the database. The relational database enabled us to quickly access all original variable names, labels, response options, and value labels from each trial for every harmonized name. Working measure by measure, exact wordings of variable labels, response options, and value labels were selected for each harmonized variable. On average, this step took approximately 7.5 hours per measure, with 109 measures harmonized (792 hours total). Work was predominantly done by ARR, in consultation with DHB and KGB. Time depended on the number of items per measure, the number of trials that used that measure, the degree of similarity between wordings used across studies, and measure modifications (e.g., added items, excluded items, changed item orders). The study team met weekly to consensus code discrepancies and select variable labels, response options, and value labels most consistent with published literature.

#### Simplifying and harmonizing across similar measures (construct by construct)

Measures were categorized according to the constructs they measured to facilitate cross-measure harmonization within each domain and to align with categories of previously assessed treatment predictors and moderators (Figure 1). Domains are outlined in Table 3 and include (1) demographics, (2) family factors (family mental health history, family accommodation, family functioning), (3) treatment factors (treatment history, concomitant treatment, CBT adherence/fidelity), (4) comorbidity (structured diagnostic interview, externalizing symptoms including inattention and hyperactivity, mood symptoms, anxiety symptoms), (5) OCD symptoms (severity, parent/child report, OCD impairment), and (6) global functioning/disability (global improvement, global severity, quality of life/disability). For trials that had multiple measures of the same construct, measures were prioritized according to how often they were used across all trials. Measures were dropped if they were used in <3 trials or did not measure one of the included constructs. If a trial used a unique measure of a construct that it did not otherwise assess, we retained the measure despite it not being used in other trials. We also retained different versions of the same measure (e.g., CDI versus CDI-2) as separate variables for later harmonization. Demographics and medical/family history were asked in survey formats (i.e., questions are qualitatively distinct and not intended to be combined into scale scores) that differed vastly across trials, precluding conventional harmonization strategies. For example, the way medications were reported ranged from listing every past and current medication by name and dosage, to listing the medication classes, to a single item indicating whether a participant had ever taken medication. To address this incredible between-study variability, we treated this process as a chart review, where we first inventoried the type of information provided by the trials, then identified key information (e.g., the specificity of medication classes) through consensus meetings, created a template of information we wanted (i.e., variable name, variable label, value labels), and finally abstracted information by trial. The abstraction process also involved coding (e.g., how medications were assigned to classes), which was done in consensus.

**Table 3.**
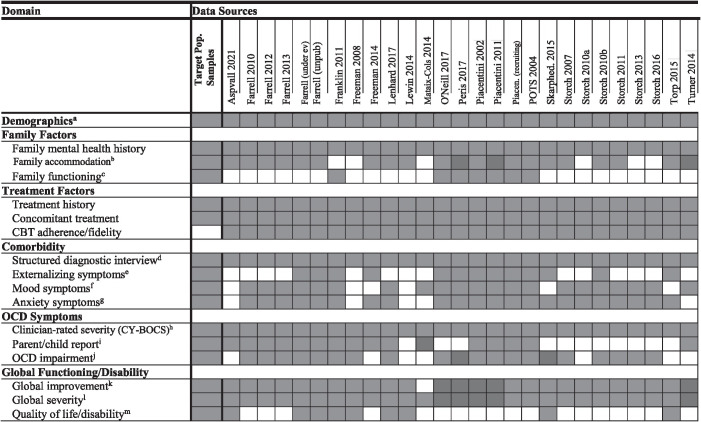
Harmonization of trial data

*Note:* Measures were collected at baseline and post-treatment (at minimum) in each trial

a Demographics include age, gender, race, ethnicity, income, education;

b Family Accommodation measured with the Family Accommodation Scale (FAS; parent report);

c Family functioning measured with parent/child report on the Family Assessment Device (FAD), Family Assessment Measure (FAM), Family Adaptability and Cohesion Evaluation Scale (FACES), and/or Family Environment Scale (FES);

d Diagnostic Interviews were clinician-administered using the Mini International Neuropsychiatric Interview for Children and Adolescents (MINI-Kid), Anxiety Disorders Interview Schedule (ADIS), or Schedule for Affective Disorders and Schizophrenia for School-Age Children (KSADS);

e Externalizing symptoms measured with parent report on the Child Behavior Checklist (CBCL) or Conner’s Parent Rating Scale (CPRS);

f Mood symptoms measured with child-report on the Child Depression Inventory (CDI), Beck Depression Inventory-Youth (BDI-Y), or Children’s Depression Rating Scale (CDRS);

g Anxiety symptoms measured with child and parent report on the Multidimensional Anxiety Scale for Children (MASC), Screen for Child Anxiety Related Disorders (SCARED) and/or Spence Child Anxiety Scale (SCAS);

h Clinician-rated OCD severity measured with the Children’s Yale-Brown Obsessive Compulsive Scale (CY-BOCS);

i child and parent report of OCD symptoms measured with the Obsessive-Compulsive Inventory (OCI) and/or CY-BOCS self-report;

j OCD impairment measured with parent and child report Child OCD Impact Scale (COIS);

k Global improvement measured with clinician report on the Clinical Global Impression-Improvement Scale (CGI-I);

l Global Severity measured with clinician report on the Clinical Global Impression-Severity Scale (CGI-S) or Children’s Global Assessment Scale (CGAS);

m Quality of Life measured with parent and child report on the Children’s Sheehan Disability Scale (CSDS), Education, Work and Social Adjustment Scale (EWSAS), Pediatric Quality of Life Enjoyment and Satisfaction Questionnaire (PQLESQ), Pediatric Quality of Life Inventory (PedsQL), and/or Questionnaire for Measuring Health-Related Quality of Life in Children and Adolescents (KINDL)

During weekly team meetings, determinations were made at the measure and item level about which variables to keep/drop across studies; any measures that were dropped in the process will be reported on in subsequent manuscripts. Harmonization of measures within each of the 10 domains took an average of 51 hours (~5.5 hours per measure; ~643 hours total). Work was done by ARR with weekly collaboration meetings with DHB and additional weekly consensus meetings with DHB, KGB, and LAN. Challenges encountered at this stage related to between-trial differences in the structure and content of the demographics and medical, medication, treatment, and family history. In particular, studies differed in the amount of information collected, how it was recorded (i.e., numeric, categorical, open-ended text response), level of specificity (e.g., father versus paternal family, medication names versus classes), number of reporters, and how reporters were identified (e.g., primary caregiver, mother, father, other), and definitions of demographic information across countries. After this reduction and simplification process, the harmonized data dictionary included 3,657 variables.

#### Wrangling IPD (trial by trial)

Working trial by trial, datasets were restructured so that each variable had the same name across assessments, resulting in multiple lines of data for the same participant with an index variable for assessment point. Restructured data retained the original variable naming for each trial. Using crosswalks developed previously, we wrote syntax to change variable names, variable labels, value options, and value labels. Scoring of measures was checked and syntax was generated so that measures had the same scoring across trials. Syntax was also generated to implement the coding of demographic, medical, and family history data. On average, this process took 52 hours per trial (range 11–92 hours; ~1,124 hours total) and was especially time-consuming due to generating unique syntax for each trial and discrepancies between the original data and corresponding data dictionaries. In addition, it was time-consuming to code the differently structured demographic, medical, and family history information. It was also challenging to investigate how various measures were scored, as different versions, item numbers, and scoring conventions were used across trials for the same measure. Work was predominantly done by ARR, guided by DHB, and with additional input from KGB.

#### Pulling it all together

As of manuscript submission, we are continuing to wrangle the 24 trials. Together the process took about 125 hours per trial, for a total of approximately 3,000 hours. This estimate was considerably higher than what was projected during the planning stages, which further emphasizes that harmonization of multiple variables and domains across trials is a time-consuming process that requires sufficient investment of time and money. Quality control was implemented by DHB checking work performed by ARR, including all harmonized variables and data screening to ensure that variables were within expected ranges. The iterative nature of harmonization means that quality control by multiple members of the team is built into the process.

### Data coding

We will use the Cochrane Revised Risk of Bias tool (RoB 2.0) to evaluate the risk of bias within each trial. Decisions about the inclusion of trials identified as being at elevated risk of bias will be made in consensus with the core research team and relevant trial investigators and partners. We will perform sensitivity analyses where trials with elevated risk of bias will be included and excluded from analyses. Information about each trial will be coded in accordance with Preferred Reporting Items for Systematic Reviews and Meta-Analyses (PRISMA) guidelines. We will use Covidence to organize and systematically review information about trial design and implementation. This work will be more involved than that of conventional meta-analysis, as we have access to the trial manuals and other documentation beyond those reported in peer-reviewed manuscripts. Trial manuals, study protocols, and primary outcome manuscripts were reviewed to inform the development of a coding template (Guest, MacQueen, & Namey, 2012) aligned with PRISMA guidelines but including more detail about treatment type and implementation to better categorize differences in treatment approaches across trials. This coding template was then reviewed with the core study team, which included experts in pediatric OCD treatment, and updated to reflect key treatment components. Codes were informed by treatment variations in clinical trials reported on in previous meta-analyses (“top-down”) and from codes inductively generated following a review of every treatment manual (“bottom-up”) by the primary coder (LAN). Codes included general information on treatment format (group, individual), mode of delivery (in person, online), treatment duration (number of total sessions, session duration, number of sessions with in-session exposure, weekly/biweekly meetings, etc.), the inclusion of core CBT components (psychoeducation, cognitive restructuring, relaxation, rewards, relapse prevention), and degree of family involvement (type, targeting of accommodation) that will be used to inform subgroup analyses. Once the template is finalized, each trial’s documentation will be double-coded by two independent coders. Codes will be compared and discrepancies resolved recursively, with input from trial investigators as needed.

### Analytic approach

To transport treatment effects from trial samples to target populations, there are required identifiability conditions and estimators. This work is based on the potential outcomes framework, which posits that each participant has an unrealized or potential outcome under each of the treatment conditions (Peterson & van der Laan, 2014; Rubin, 2005). In a trial, one potential outcome, at most, may be observed for each individual (because they are assigned to one treatment), while the other potential outcomes (treatments to which the individual is not assigned) remain unobserved (counterfactual). The causal parameter that will allow comparisons among interventions evaluated in different trials is the potential outcome mean (POM), which is an estimate of how the target population is expected to respond, had they received the intervention. Generating unbiased estimates of POMs requires making identifiability assumptions, building accurate statistical models to adjust for covariates, and protecting against measured confounding. The identifiability assumptions include within-trial assumptions common to causal inference using RCT designs (consistency, exchangeability of treatment conditions, positivity) and between-trial assumptions that mirror the within-trial assumptions (consistency of potential outcomes across trials, exchangeability of trial participation, and positivity of trial participation; (Barker et al., 2022; Barker et al., 2023). These assumptions imply that after adjusting for covariates, there is no confounding that links the outcome with either trial selection or treatment assignment (Dahabreh et al., 2019; Dahabreh et al., 2020). When transporting POMs, the covariates of interest are variables that either predict the outcome or modify treatment response and that relate to trial participation or treatment assignment. In other words, to generalize a POM from one sample to another, between-trial differences in baseline characteristics affecting treatment response must be measured and adjusted using statistical modeling. If all such covariates are measured and models are accurate, then resulting estimates are unbiased and POMs can be safely transported to a sample of the target population (Barker et al., 2022; Barker et al., 2023; Dahabreh et al., 2019; Dahabreh et al., 2020).

Importantly, identifiability conditions required for causal inference when pooling data across trials are not unique to transportability analysis and are shared with conventional meta-analytic approaches, although they are rarely made explicit. Advances in transportability analysis can capitalize on IPD from each trial and the target population to make weaker assumptions than those implicitly required by conventional meta-analysis. For example, identifiability assumptions can hold conditional on covariates, and doubly robust estimators can be used to provide more robust statistical estimates (Barker et al., 2023). The approach also justifies making inferences in populations that differ from the trial populations and making novel comparisons among treatments within subpopulations using unbiased estimates of POMs for each treatment in the target population or subpopulations. The advantages of transportability methods over meta-analysis and individual participant data meta-analysis are summarized in Table 1.

#### Generate comparative effectiveness estimates in the target population

Generating comparative effectiveness estimates in the target population requires identifying key covariates, building working models (i.e., predictive models that are part of a statistical estimator), using the models to transport estimates from trials to the target population, and performing subgroup analyses.

**Identifying covariates.** Covariates will include treatment modifiers identified by previous literature (see Figure 1). One of the limitations in the pediatric OCD literature is that within-trial sample sizes do not support robust evaluation of treatment modifiers. Pooling data across trials can help overcome this limitation, but only for variables that were assessed in multiple trials of the same treatment. There is thus a tension between evaluating a wide range of possible covariates within single trials and evaluating a more limited number in a pooled sample. We will use two data-adaptive approaches to variable selection. First, we will integrate the pooled dataset using regularized linear models (i.e., Lasso). This approach will include covariates included in a majority of the trials. The second approach will again use Lasso models with data from the larger trials, which will enable us to include a larger number of potential covariates.

**Building working models.** Two types of models are used in causal estimators of treatment effects in the target population: models that predict the outcome and models that predict trial assignment (e.g., propensity scores). It is also possible to use both types of working models to provide a doubly robust estimator that is unbiased if either of the two types of models is correctly specified (Dahabreh et al., 2019; Dahabreh et al., 2020). Within each subset of trials that evaluated the same treatment, we will use Lasso models to build both prediction and trial assignment models to enable the use of the double robust estimators.

**Transporting estimates.** Once working models are built for each type of treatment, we will fit a doubly robust causal estimator to estimate POMs for the target population (Barker et al., 2023; Dahabreh et al., 2019; Dahabreh et al., 2020). Essentially, the baseline covariates from the target population are used in the working models to generate an estimate of what the POM would be, had the target population received the intervention, given the modeled covariates. Average treatment effects will be estimated by taking the difference between POMs. Non-parametric bootstrap sampling will be used to generate standard errors, with participants sampled with replacement from each of the trial/treatment arms. The accuracy of the transported estimates will be evaluated using a type of cross-validation where the observed treatment outcomes in each trial are used as the ground truth for other trials that evaluated the same treatment. For each treatment, each trial will be iteratively set aside and used as the target population for the other trials.

**Subgroup analysis.** Although other possible modifiers will be explored, POMs will be compared for each treatment condition among subsamples defined by indicators examined in previous meta-analyses, including demographics, family factors, treatment factors, comorbidity, OCD symptoms, and global functioning/disability. Estimates within subsamples are estimated using the same working models as the overall estimates but then marginalizing over those who belong to a subsample instead of marginalizing over the entire target population sample. Average treatment effects will be calculated within each sub-sample. Because the comparative effectiveness estimates within target subsamples use the same working models as the overall target sample, the analytics are a natural extension of those described above. The challenge is in understanding the implications and nuances of how treatments interact with subpopulations. For example, some treatments might not be appropriate for some subsamples (younger children). To address this challenge, the research team will work closely with the trial investigators and partners to ensure that relevant features of the treatment are well understood in the context of treating the subpopulation of interest.

**Missing data**. Two sources of missing data are expected: (1) within-trial missingness due to drop-out or missed assessments (expected 10%–20%; Johnco et al., 2020) and (2) between-trial or systematic missing data due to differences in assessment battery. Fortunately, pediatric OCD RCTs have been using the CY-BOCS for some time, which will provide a consistent outcome and baseline symptom severity measure. Trials also used common diagnostic interviews with strong normative data, enabling us to equate the presence of comorbidities using clinical cut-offs. Other clinical measures will also be harmonized using normative data. We will address the remaining systematic missing data using current recommendations for CI-MA (Steingrimsson et al., 2024).

### Dissemination

#### Partner involvement

During pre-harmonization, the Dissemination Partner group met twice to review project objectives, define group member roles, and identify any additional partners. During post-harmonization, the group will meet twice to (1) review the list of comparative variables that result from harmonization, (2) provide input on planned analyses to ensure utility of findings (Brownson et al., 2013; Concannon et al., 2019; Noar, Harrington, & Aldrich, 2009), and (3) discuss potential dissemination streams within IOCDF’s communication tool network. During post-analyses/dissemination, the group will use feedback to iteratively improve dissemination content and strategy. Potential strategies for disseminating study findings include (1) blog and social media posts co-developed with the dissemination partner group to identify potential audiences (e.g., patients/families, providers, policymakers) and design content best targeted towards these audiences, (2) leverage outlets like IOCDF to present findings in a variety of formats (e.g., newsletter, IOCDF social media, etc.), and (3) present at IOCDF conferences that are geared towards both research and clinical (e.g., patient/families) audiences. We will continue to hone our dissemination strategy and work with our partners to explore other unique avenues. Partner suggestions to date include creating a graphic novel, a brief animated video, and music.

#### Publicly available data and code

Our team will facilitate the upload of de-identified harmonized datasets to the NIMH NDA and additional resources (e.g., regression models, analytic syntax) that will allow individuals to replicate study analyses within other target populations. Because data in target populations were not collected under controlled settings, they may not be a good fit for the NDA; if not accepted, data will be made available as part of published manuscripts and/or upon request.

#### Future clinical decision-making tools

CI-MA has distinct advantages for the development of decision tools to help match patients to optimal treatment, as this approach, unlike other meta-analytic methods, can compare interventions evaluated in different settings, with different treatment and control arms, and among different trial populations (e.g., older samples, more severe symptoms, different comorbidity mixtures). As a result, code from Project Harmony can be used to transport original study findings into new and different settings (e.g., intensive, outpatient) and for new end users. The methods also maximize information from subgroups typically under-represented in RCTs (i.e., different races, ethnicities, and co-occurring autism diagnoses), for which there is not enough data in any given trial to draw substantive conclusions. Thus, when completed, Project Harmony will be the best available dataset and analytic code upon which to build a precision intervention clinical decision-making tool for youth with OCD.

## Discussion

Our experience to date with acquiring and harmonizing IPD from RCTs in pediatric OCD reinforces the idea that careful planning and understanding of harmonization is needed to accurately budget project time and resources; it is easy to be overly optimistic about the projected effort required. Project Harmony will also use data adaptive approaches to identify treatment modifiers, which will be used together with CI-MA to provide the best comparative effectiveness estimates given current evidence, including comparisons among treatments that have not been evaluated in head-to-head trials. These estimates will be generated for target populations and important subpopulations within pediatric OCD. When completed, this analysis will provide the best evidence available for what works for whom in pediatric OCD. Finally, we will coordinate with partners to facilitate the dissemination of study findings, enabling future work extending inferences to other pediatric OCD populations and developing clinical decision-making tools. Importantly, the outlined approach can also be utilized to better answer the question “What works, for whom?” with greater specificity across a range of disorders, facilitating a more personalized approach to treatment.

## References

[r1] Aspvall, K., Andersson, E., Melin, K., Norlin, L., Eriksson, V., Vigerland, S., … & Serlachius, E. (2021). Effect of an internet-delivered stepped-care program vs in-person cognitive behavioral therapy on obsessive-compulsive disorder symptoms in children and adolescents: A randomized clinical trial. JAMA, 325(18), 1863–1873. 33974020 10.1001/jama.2021.3839 PMC8114140

[r3] Barker, D. H., Dahabreh, I. J., Steingrimsson, J. A., Houck, C., Donenberg, G., DiClemente, R.,& Brown, L. K. (2022). Causally interpretable meta-analysis: Application in adolescent HIV prevention. Prevention Science, 1–12. 10.1007/s11121-021-01270-3 PMC8742835 34241752

[r4] Barker, D. H., Bie, R., & Steingrimsson, J. A. (2023). Addressing systematic missing data in the context of causally interpretable meta-analysis. Prevention Science, 24(8), 1648–1658. 37726579 10.1007/s11121-023-01586-2

[r5] Brownson, R. C., Jacobs, J. A., Tabak, R. G., Hoehner, C. M., & Stamatakis, K. A. (2013). Designing for dissemination among public health researchers: Findings from a national survey in the United States. American Journal of Public Health, 103(9), 1693–1699. 23865659 10.2105/AJPH.2012.301165 PMC3966680

[r6] Cervin, M., McGuire, J. F., D’Souza, J. M., De Nadai, A. S., Aspvall, K., Goodman, W. K., … & Storch, E. A. (2024). Efficacy and acceptability of cognitive-behavioral therapy and serotonin reuptake inhibitors for pediatric obsessive-compulsive disorder: A network meta-analysis. Journal of Child Psychology and Psychiatry 65(5), 594–609. 38171647 10.1111/jcpp.13934

[r8] Chorpita, B. F., & Daleiden, E. L. (2009). Mapping evidence-based treatments for children andadolescents: Application of the distillation and matching model to 615 treatments from 322 randomized trials. Journal of Consulting and Clinical Psychology, 77(3), 566. 19485596 10.1037/a0014565

[r9] Concannon, T. W., Grant, S., Welch, V., Petkovic, J., Selby, J., Crowe, S., … & Multi Stakeholder Engagement (MuSE) Consortium. (2019). Practical guidance for involving stakeholders in health research. Journal of General Internal Medicine, 34, 458–463. 30565151 10.1007/s11606-018-4738-6 PMC6420667

[r10] Curran, P. J., & Hussong, A. M. (2009). Integrative data analysis: The simultaneous analysis ofmultiple data sets. Psychological Methods, 14(2), 81. 19485623 10.1037/a0015914 PMC2777640

[r11] Dahabreh, I. J., Robertson, S. E., Tchetgen, E. J., Stuart, E. A., & Hernán, M. A. (2019). Generalizing causal inferences from individuals in randomized trials to all trial-eligible individuals. Biometrics, 75(2), 685–694. 30488513 10.1111/biom.13009 PMC10938232

[r12] Dahabreh, I. J., Petito, L. C., Robertson, S. E., Hernán, M. A., & Steingrimsson, J. A. (2020). Towards causally interpretable meta-analysis: Transporting inferences from multiple randomized trials to a new target population. Epidemiology (Cambridge, Mass.), 31(3), 334. 32141921 10.1097/EDE.0000000000001177 PMC9066547

[r13] Farrell, L. J., Schlup, B., & Boschen, M. J. (2010). Cognitive–behavioral treatment of childhood obsessive–compulsive disorder in community-based clinical practice: Clinical significance and benchmarking against efficacy. Behaviour Research and Therapy, 48 *(*5), 409–417. 20181328 10.1016/j.brat.2010.01.004

[r57] Farrell, L., Waters, A., Milliner, E., & Ollendick, T. (2012). Comorbidity and treatment response in pediatric obsessive-compulsive disorder: a pilot study of group cognitive-behavioral treatment. Psychiatry research, 199(2), 115–123. 22633155 10.1016/j.psychres.2012.04.035

[r14] Farrell, L. J., Waters, A. M., Boschen, M. J., Hattingh, L., McConnell, H., Milliner, E. L., … & Storch, E. A. (2013). Difficult-to-treat pediatric obsessive-compulsive disorder: Feasibility and preliminary results of a randomized pilot trial of D-cycloserine-augmented behavior therapy. Depression and Anxiety, 30(8), 723–731. 23722990 10.1002/da.22132

[r15] Farrell, L. J., Oar, E. L., Waters, A. M., McConnell, H., Tiralongo, E., Garbharran, V., & Ollendick, T. (2016). Brief intensive CBT for pediatric OCD with E-therapy maintenance. Journal of Anxiety Disorders, 42, 85–94. 27395805 10.1016/j.janxdis.2016.06.005

[r58] Farrell, L. J., Waters, A. M., Tiralongo, E., Mathieu, S., McKenzie, M., Garbharran, V., Ware, R. S., Zimmer-Gembeck, M. J., McConnell, H., Lavell, C., Cadman, J., Ollendick, T. H., Hudson, J. L., Rapee, R. M., McDermott, B., Geller, D., & Storch, E. A. (2022). Efficacy of D-cycloserine augmented brief intensive cognitive-behavioural therapy for paediatric obsessive-compulsive disorder: A randomised clinical trial. Depression and anxiety, 39(6), 461–473. 10.1002/da.23242 35084071 PMC9303435

[r59] Freeman, J. B., Garcia, A. M., Coyne, L., Ale, C., Przeworski, A., Himle, M., Compton, S., & Leonard, H. L. (2008). Early childhood OCD: preliminary findings from a family-based cognitive-behavioral approach. Journal of the American Academy of Child and Adolescent Psychiatry, 47(5), 593–602. 10.1097/CHI.0b013e31816765f9 18356758 PMC2820297

[r16] Franklin, M. E., Sapyta, J., Freeman, J. B., Khanna, M., Compton, S., Almirall, D., … & March, J. S. (2011). Cognitive behavior therapy augmentation of pharmacotherapy in pediatric obsessive-compulsive disorder: The Pediatric OCD Treatment Study II (POTS II) randomized controlled trial. JAMA, 306(11), 1224–1232. 21934055 10.1001/jama.2011.1344 PMC3495326

[r17] Freeman, J., Sapyta, J., Garcia, A., Compton, S., Khanna, M., Flessner, C., … & Franklin, M. (2014). Family-based treatment of early childhood obsessive-compulsive disorder: The Pediatric Obsessive-Compulsive Disorder Treatment Study for Young Children (POTS Jr)—A randomized clinical trial. JAMA Psychiatry, 71(6), 689–698. 24759852 10.1001/jamapsychiatry.2014.170 PMC4511269

[r18] Freeman, J., Benito, K., Herren, J., Kemp, J., Sung, J., Georgiadis, C., … & Garcia, A. (2018). Evidence base update of psychosocial treatments for pediatric obsessive-compulsive disorder: Evaluating, improving, and transporting what works. Journal of Clinical Child & Adolescent Psychology, 47(5), 669–698. 30130414 10.1080/15374416.2018.1496443

[r19] Guest, G., MacQueen, K. M., & Namey, E. E. (2012). Applied thematic analysis. SAGE.

[r20] Guy, W. (1976). Clinical global impressions scale. PsycTESTS Dataset. 10.1037/t48216

[r21] Grimshaw, J. M., Eccles, M. P., Lavis, J. N., Hill, S. J., & Squires, J. E. (2012). Knowledgetranslation of research findings. Implementation Science, 7(1), 1–17. 10.1186/1748-5908-7-50 PMC3462671 22651257

[r22] Hernan, M. A., & Robins, J. M. (2010). Causal Inference: What If. Chapman and Hall/CRC.

[r23] Hothorn, T., & Zeileis, A. (2015). partykit: A modular toolkit for recursive partytioning in R. The Journal of Machine Learning Research, 16(1), 3905–3909.

[r24] Johnco, C., McGuire, J. F., Roper, T., & Storch, E. A. (2020). A meta-analysis of dropout ratesfrom exposure with response prevention and pharmacological treatment for youth with obsessive compulsive disorder. Depression and Anxiety, 37(5), 407–417. 31778595 10.1002/da.22978

[r25] Kemp, J., Barker, D., Benito, K., Herren, J., & Freeman, J. (2021). Moderators of psychosocial treatment for pediatric obsessive-compulsive disorder: Summary and recommendations for future directions. Journal of Clinical Child & Adolescent Psychology, 50(4), 478–485. 32706265 10.1080/15374416.2020.1790378

[r26] Kothari, A., McCutcheon, C., & Graham, I. D. (2017). Defining integrated knowledge translation and moving forward: A response to recent commentaries. International Journal of Health Policy and Management, 6(5), 299. 28812820 10.15171/ijhpm.2017.15 PMC5417154

[r27] Lenhard, F., Andersson, E., Mataix-Cols, D., Rück, C., Vigerland, S., Högström, J., … & Serlachius, E. (2017). Therapist-guided, internet-delivered cognitive-behavioral therapy for adolescents with obsessive-compulsive disorder: A randomized controlled trial. Journal of the American Academy of Child & Adolescent Psychiatry, 56(1), 10–19. 27993223 10.1016/j.jaac.2016.09.515

[r28] Mataix-Cols, D., Turner, C., Monzani, B., Isomura, K., Murphy, C., Krebs, G., & Heyman, I. (2014). Cognitive–behavioural therapy with post-session D-cycloserine augmentation for paediatric obsessive–compulsive disorder: Pilot randomised controlled trial. The British Journal of Psychiatry, 204(1), 77–78. 24262813 10.1192/bjp.bp.113.126284

[r29] McGuire, J. F., Piacentini, J., Lewin, A. B., Brennan, E. A., Murphy, T. K., & Storch, E. A.(2015). A meta-analysis of cognitive behavior therapy and medication for child obsessive–compulsive disorder: Moderators of treatment efficacy, response, and remission. Depression and Anxiety, 32(8), 580–593. 26130211 10.1002/da.22389 PMC4515191

[r30] Noar, S. M., Harrington, N. G., & Aldrich, R. S. (2009). The role of message tailoring in thedevelopment of persuasive health communication messages. Annals of the International Communication Association, 33(1), 73–133.

[r31] O’Neill, J., Piacentini, J., Chang, S., Ly, R., Lai, T. M., Armstrong, C. C., … & Nurmi, E. L. (2017). Glutamate in pediatric obsessive-compulsive disorder and response to cognitive-behavioral therapy: Randomized clinical trial. Neuropsychopharmacology, 42(12), 2414–2422. 28409563 10.1038/npp.2017.77 PMC5645751

[r32] Öst, L. G., Riise, E. N., Wergeland, G. J., Hansen, B., & Kvale, G. (2016). Cognitive behavioral and pharmacological treatments of OCD in children: A systematic review and meta-analysis. Journal of Anxiety Disorders, 43, 58–69. 27632568 10.1016/j.janxdis.2016.08.003

[r33] Pearl, J., & Bareinboim, E. (2014). External validity: From do-calculus to transportability across populations. Statistical Science, 29(4), 579–595.

[r34] Peris, T. S., Rozenman, M. S., Sugar, C. A., McCracken, J. T., & Piacentini, J. (2017). Targeted family intervention for complex cases of pediatric obsessive-compulsive disorder: A randomized controlled trial. Journal of the American Academy of Child & Adolescent Psychiatry, 56(12), 1034–1042. 29173737 10.1016/j.jaac.2017.10.008 PMC5875916

[r35] Petersen, M. L., & van der Laan, M. J. (2014). Causal models and learning from data: Integrating causal modeling and statistical estimation. Epidemiology (Cambridge, Mass.), 25(3), 418. 24713881 10.1097/EDE.0000000000000078 PMC4077670

[r36] Piacentini, J., Peris, T., Bergman, R.L., Chang, S., & Jaffer, M. (2007). BRIEF REPORT: Functional impairment in childhood OCD: Development and psychometrics properties of the child obsessive-compulsive impact scale-revised (COIS-R), Journal of Clinical Child and Adolescent Psychology, 36(4), 645–653. 18088221 10.1080/15374410701662790

[r37] Piacentini, J., Bergman, R. L., Jacobs, C., McCracken, J. T., & Kretchman, J. (2002). Open trial of cognitive behavior therapy for childhood obsessive–compulsive disorder. Journal of Anxiety Disorders, 16(2), 207–219. 12194545 10.1016/s0887-6185(02)00096-8

[r38] Piacentini, J., Bergman, R. L., Chang, S., Langley, A., Peris, T., Wood, J. J., & McCracken, J. (2011). Controlled comparison of family cognitive behavioral therapy and psychoeducation/relaxation training for child obsessive-compulsive disorder. Journal of the American Academy of Child & Adolescent Psychiatry, 50(11), 1149–1161. 22024003 10.1016/j.jaac.2011.08.003 PMC3205429

[r39] Rubin, D. B. (2005). Causal inference using potential outcomes: Design, modeling,decisions. Journal of the American Statistical Association, 100(469), 322–331.

[r40] Scahill, L., Riddle, M. A., McSwiggan-Hardin, M., Ort, S. I., King, R. A., Goodman, W. K., et al. (1997). Children’s Yale-Brown Obsessive-Compulsive Scale: Reliability and validity. Journal of the American Academy of Child and Adolescent Psychiatry, 36, 844–852. 9183141 10.1097/00004583-199706000-00023

[r42] Skarphedinsson, G., Weidle, B., Thomsen, P. H., Dahl, K., Torp, N. C., Nissen, J. B., … & Ivarsson, T. (2015). Continued cognitive-behavior therapy versus sertraline for children and adolescents with obsessive–compulsive disorder that were non-responders to cognitive-behavior therapy: A randomized controlled trial. European Child & Adolescent Psychiatry, 24, 591–602. 25239489 10.1007/s00787-014-0613-0 PMC4419185

[r43] Steingrimsson, J. A., Barker, D. H., Bie, R., & Dahabreh, I. J. (2024). Systematically missing data in causally interpretable meta-analysis. Biostatistics, 25(2), 289–305. 36977366 10.1093/biostatistics/kxad006 PMC11017122

[r62] Steingrimsson, J. A., Wen, L., Voter, S., & Dahabreh, I. J. (2024). Interpretable meta-analysis of model or marker performance. arXiv preprint arXiv:2409.13458.

[r44] Storch, E. A., Geffken, G. R., Merlo, L. J., Mann, G., Duke, D., Munson, M., … & Goodman, W. K. (2007). Family-based cognitive-behavioral therapy for pediatric obsessive-compulsive disorder: Comparison of intensive and weekly approaches. Journal of the American Academy of Child & Adolescent Psychiatry, 46(4), 469–478. 17420681 10.1097/chi.0b013e31803062e7

[r45] Storch, E. A., Lehmkuhl, H. D., Ricketts, E., Geffken, G. R., Marien, W., & Murphy, T. K. (2010a). An open trial of intensive family based cognitive-behavioral therapy in youth with obsessive-compulsive disorder who are medication partial responders or nonresponders. Journal of Clinical Child & Adolescent Psychology, 39(2), 260–268. 10.1080/15374410903532676 20390817

[r46] Storch, E. A., Murphy, T. K., Goodman, W. K., Geffken, G. R., Lewin, A. B., Henin, A., Micco, J. A., Sprich, S., Wilhelm, S., Bengtson, M. & Geller, D. A. (2010b). A preliminary study of D-cycloserine augmentation of cognitive-behavioral therapy in pediatric obsessive-compulsive disorder. Biological Psychiatry, 68(11), 1073–1076. 20817153 10.1016/j.biopsych.2010.07.015 PMC3034091

[r47] Storch, E. A., Caporino, N. E., Morgan, J. R., Lewin, A. B., Rojas, A., Brauer, L., … & Murphy, T. K. (2011). Preliminary investigation of web-camera delivered cognitive-behavioral therapy for youth with obsessive-compulsive disorder. Psychiatry Research, 189(3), 407–412. 21684018 10.1016/j.psychres.2011.05.047

[r48] Storch, E. A., Bussing, R., Small, B. J., Geffken, G. R., McNamara, J. P., Rahman, O., … & Murphy, T. K. (2013). Randomized, placebo-controlled trial of cognitive-behavioral therapy alone or combined with sertraline in the treatment of pediatric obsessive–compulsive disorder. Behaviour Research and Therapy, 51(12), 823–829. 24184429 10.1016/j.brat.2013.09.007 PMC3908957

[r49] Storch, E. A., Wilhelm, S., Sprich, S., Henin, A., Micco, J., Small, B. J., … & Geller, D. A. (2016). Efficacy of augmentation of cognitive behavior therapy with weight-adjusted d-cycloserine vs placebo in pediatric obsessive-compulsive disorder: A randomized clinical trial. JAMA Psychiatry, 73(8), 779–788. 27367832 10.1001/jamapsychiatry.2016.1128 PMC5734635

[r50] Strobl, C., Boulesteix, A. L., Zeileis, A., & Hothorn, T. (2007). Bias in random forest variableimportance measures: Illustrations, sources and a solution. BMC Bioinformatics, 8(1), 1–21. 17254353 10.1186/1471-2105-8-25 PMC1796903

[r51] POTS Team (2004). Cognitive-behavior therapy, sertraline, and their combination for children and adolescents with obsessive-compulsive disorder: The Pediatric OCD Treatment Study (POTS) randomized controlled trial. JAMA, 292(16), 1969–1976. 15507582 10.1001/jama.292.16.1969

[r52] Torp, N. C., Dahl, K., Skarphedinsson, G., Thomsen, P. H., Valderhaug, R., Weidle, B., … & Ivarsson, T. (2015). Effectiveness of cognitive behavior treatment for pediatric obsessive-compulsive disorder: Acute outcomes from the Nordic Long-term OCD Treatment Study (NordLOTS). Behaviour Research and Therapy, 64, 15–23. 25463245 10.1016/j.brat.2014.11.005

[r53] Turner, C., O’Gorman, B., Nair, A., & O’Kearney, R. (2018). Moderators and predictors of response to cognitive behaviour therapy for pediatric obsessive-compulsive disorder: A systematic review. Psychiatry Research, 261, 50–60. 29287236 10.1016/j.psychres.2017.12.034

[r63] Turner, C. M., Mataix-Cols, D., Lovell, k., Krebs, G., Lang, K., Byford, S., & Heyman, I. (2014). Telephone Cognitive-Behavioral Therapy for Adolescents With Obsessive-Compulsive Disorder: A Randomized Controlled Non-inferiority Trial. Journal of the American Academy of Child & Adolescent Psychiatry, 53(12), 1298–1307.e2. 10.1016/j.jaac.2014.09.012 25457928 PMC4305192

[r54] Valderhaug, R., & Ivarsson, T. (2005). Functional impairment in clinical samples of Norwegian and Swedish children and adolescents with obsessive-compulsive disorder. European Child & Adolescent Psychiatry, 14, 164–173. 15959662 10.1007/s00787-005-0456-9

[r55] Williams, M., Powers, M., Yun, Y. G., & Foa, E. (2010). Minority participation in randomized controlled trials for obsessive–compulsive disorder. Journal of Anxiety Disorders, 24(2), 171. 20143498 10.1016/j.janxdis.2009.11.004 PMC3431431

[r56] Woolf, S. H., Zimmerman, E., Haley, A., & Krist, A. H. (2016). Authentic engagement of patients and communities can transform research, practice, and policy. Health Affairs, 35(4), 590–594. 27044956 10.1377/hlthaff.2015.1512 PMC4868544

